# Development of a Quantitative Food Frequency Questionnaire for Use among the Yup'ik People of Western Alaska

**DOI:** 10.1371/journal.pone.0100412

**Published:** 2014-06-25

**Authors:** Fariba Kolahdooz, Desiree Simeon, Gary Ferguson, Sangita Sharma

**Affiliations:** 1 Department of Medicine, University of Alberta, Edmonton, Alberta, Canada; 2 Alaska Native Tribal Health Consortium, Community Health Services Division, Wellness and Prevention Department, Anchorage, Alaska, United States of America; Oklahoma State University, United States of America

## Abstract

Alaska Native populations are experiencing a nutrition transition and a resulting decrease in diet quality. The present study aimed to develop a quantitative food frequency questionnaire to assess the diet of the Yup'ik people of Western Alaska. A cross-sectional survey was conducted using 24-hour recalls and the information collected served as a basis for developing a quantitative food frequency questionnaire. A total of 177 males and females, aged 13-88, in six western Alaska communities, completed up to three 24-hour recalls as part of the Alaska Native Dietary and Subsistence Food Assessment Project. The frequency of the foods reported in the 24-hour recalls was tabulated and used to create a draft quantitative food frequency questionnaire, which was pilot tested and finalized with input from community members. Store-bought foods high in fat and sugar were reported more frequently than traditional foods. Seven of the top 26 foods most frequently reported were traditional foods. A 150-item quantitative food frequency questionnaire was developed that included 14 breads and crackers; 3 cereals; 11 dairy products; 69 meats, poultry and fish; 13 fruit; 22 vegetables; 9 desserts and snacks; and 9 beverages. The quantitative food frequency questionnaire contains 39 traditional food items. This quantitative food frequency questionnaire can be used to assess the unique diet of the Alaska Native people of Western Alaska. This tool will allow for monitoring of dietary changes over time as well as the identification of foods and nutrients that could be promoted in a nutrition intervention program intended to reduce chronic disease.

## Introduction

The diet of Alaska Native people has been in transition. Prior to the availability of store-bought foods, the diet consisted of locally harvested foods such as marine mammals, land mammals, ocean and freshwater fish, birds and many varieties of berries [1]. These “traditional” foods are rich sources of many nutrients, such as protein, iron, vitamins D and B12, selenium, and mono- and polyunsaturated fats [2]–[4]. The current diet of Alaska Native people contains a mixture of traditional foods and imported “store-bought” foods, which are often energy-dense, but of low nutritional quality [2]–[6]. Even though store-bought foods have only become widely available in Alaska in the last 60 years, the transition away from a diet composed entirely of traditional foods has been rapid. One of the first dietary assessment studies in rural Alaska, which took place from 1956–1961 [7], found that store-bought foods contributed to over 50% of energy intake [7]. More recent studies have shown that traditional foods provide 15–25% of total energy [6], [8]. Although some studies have shown that older Alaska Native people consume a higher percentage of traditional foods than younger generations [2], [6], these foods still contribute to the nutritional quality of the diet in all age groups [2], [4], [6], [9]. In twelve Alaskan communities, in 2009, traditional foods contributed 23% of energy intake, yet supplied 46% of protein, 37% of iron, 90% of eicosapentaenoic acid (EPA) and 83% of vitamin D [10].

Concurrent to the diet and lifestyle transition, Alaska Native peoples are experiencing an increase in diet-related chronic diseases [9], [11]–[15]. In the 1950's, obesity among Alaska Native people in the western region was rare and confined to age groups <60 years [16]. In 2007, the Behavioural Risk Factor Surveillance System [12] found that 28% of Alaska Native people were overweight and 34% were obese. In addition, diabetes prevalence increased between 1990 and 2006 [16] by 114% among Alaska Native people state wide, and by 152% in the western region [9]. While the mortality rate from ischemic heart disease has declined dramatically over the last 20 years among the United States Caucasian population, it has remained relatively constant in Alaska Native populations [13]. Currently, the leading cause of death among Alaska Native people is cancer [14], and cancer mortality rates among Alaska Native men and women are significantly higher than among Caucasian Americans [14], [15].

Programs that promote dietary improvements can be effective strategies to reduce the burden of chronic disease among Alaska Native people [17]–[19]. However, little information is available on the current diet of this population in terms of food, nutrient and food group intakes. Tools that can accurately capture a target population's dietary pattern can help identify foods and nutrients to be targeted in intervention programs. Further, such tools can be used in monitoring and evaluating a program's effectiveness. Food frequency questionnaires are considered the method of dietary assessment best suited for large epidemiological applications due to their relative ease of use, cost-effectiveness and ability to estimate usual intake over extended periods of time [20], [21]. To the best of our knowledge, no such detailed, up-to-date, population-specific dietary instrument has been developed for Alaska Native peoples.

The aim of this study is to describe the current diet and to develop a quantitative food frequency questionnaire (QFFQ) to assess the unique diet of Native populations of Western Alaska. The QFFQ could be used to identify foods and nutrients to be targeted in a culturally appropriate nutritional intervention, evaluate the impact of diet intervention programs, as well as monitor the nutrition transition occurring in this population.

## Methods

### Setting

The Alaska Native Dietary and Subsistence Food Assessment Project (ANDSFAP) took place in six rural communities in Western Alaska's Yukon–Kuskokwim Delta region. Participating villages ranged in size from 287 people and 83 households to 721 people and 199 households [22]. These villages are geographically remote; they are not on a road system and are accessible only by small plane or boat and by snow machine in the winter. Residents are predominantly Alaska Native people.

### Sampling

The project was presented to tribal councils and written resolutions to participate were received from six villages. Enrolment procedures followed the directive of each tribal council. Village residents aged ≥13 years were randomly recruited to participate in 24-hour recalls; the target for recruitment was 30 people per village. To aid in capturing maximum diet diversity, only one person was recruited per household. Written consent or assent was obtained from each participant and from a parent for those under 18 years of age.

### 24-hour dietary recall collection

24-hour recall data collection was undertaken prior to the current study and described previously [10]. In brief, the 24-hour recalls were obtained by trained interviewers who recorded information on food/beverages consumed in the previous 24 hours, including type, amount, brand name, food source, food processing method, food preparation method and any additions (e.g. sugar in coffee). Portion size was assessed using a variety of standard utensils and food models. A multiple pass method was used to help ensure that no items were inadvertently omitted. Follow–up interviews were conducted over the telephone to obtain any details omitted in error. To capture seasonal variation, attempts were made to interview each participant four times, once per season. The 24-hour recall interviews were conducted in English, or a combination of English and Yup'ik using an interpreter. The 24 hour recall information was used for FFQ development.

### Development of the Quantitative Food Frequency Questionnaire

The frequency of the foods reported in the 24-hour recalls was tabulated. Any food reported more than two times was included in the draft QFFQ which can be found in the supplementary data [see Table S1], with the exception of low-nutrient foods such as spices and condiments. Foods that did not appear in the 24-hour recalls but were considered relevant to the population, such as seasonal foods, were also added. Additional blank lines were provided under each food group for respondents to list any other foods or drinks they consumed. Local community members were consulted to ensure no commonly consumed foods were omitted. Foods expected to be promoted in a subsequent intervention (e.g., diet soda) were also included.

To assess the portion sizes of foods/beverages consumed, food models were carefully selected to represent serving sizes of each item on the QFFQ. Standard bowls, cups and spoons of the type and size available locally were used. Other food models were constructed by the study team and local residents using standard units familiar to participants, such as a slice of bread. Wrappers and containers from familiar foods, such as cans of vegetables and bags of chips, were also used to aid in portion estimation. Food models represented a wide range of serving sizes.

Similar food items were grouped on the QFFQ and resulting categories included breads and crackers; cereal; dairy, meat, poultry and fish; fruit; vegetables; desserts and snacks; and beverages. Food categories were ordered according to cultural preference and dietary habits based on advice and input of local community members. A manual of procedures was developed for the administration of the draft QFFQ and all staff was trained in its use. The draft QFFQ was pilot tested in a convenience sample of 15 individuals selected from men and women in one of the communities participating in the study. The purpose of this pilot was to ensure that the questionnaire was culturally appropriate and the structure and order of the foods were user-friendly for interviewers. Subsequently, because of time constraints for completion, similar items were combined. Foods reported less than six times in the original 24-hour recall data set were not included in the QFFQ due to their low overall contribution to the diet. Similar studies set a precedent for these modifications [23], [24].

This study was conducted according to the guidelines laid down in the Declaration of Helsinki and all procedures involving human subjects/patients were approved by the Alaska Area Institutional Review Board and the Office of University Research Ethics at the University of North Carolina at Chapel Hill. The project was also reviewed and approved by the Yukon-Kuskokwim Health Corporation and the Norton Sound Health Corporation. Six villages in Western Alaska gave written resolutions to participate in the project.

### Data analysis

Statistical Analysis Systems (SAS Institute, Cary, NC, USA) was used for descriptive statistical analysis of the dietary intake and manipulation of the data.

## Results

### 24-hour recalls

A total of 400 recalls were collected from 177 Yup'ik participants (73 males and 104 females) aged 13–88 for the ANDSFAP. Most participants completed two or three 24-hour recalls, 27 completed one 24-hr dietary recalls, and 35 completed all four. Table 1 lists the most commonly reported foods from the 24-hour recalls. Commercially available foods were the most predominant. Seven traditional foods were among the twenty-six most commonly reported foods. Coffee and sugar were each reported over 300 times. High-sugar drinks such as juice or flavoured drink and soda pop were frequently reported, as were white bread and rice. Table 2 lists traditional foods most frequently reported. Fish was the most common traditional food consumed (over 40%).

**Table 1:** Foods most commonly reported on 400, 24-hour recalls among Alaska Native people (n = 177).

Food	Number of times reported	Number of people reporting
Coffee, any type	400	135
Sugar	330	100
Flavoured drink	323	125
Soda pop	296	111
White bread and rolls	249	103
White rice	240	120
Tea	205	75
Potatoes	184	99
Fish other than salmon and halibut	154	80
Pilot bread crackers	144	85
King Salmon, smoked	109	76
Agutuk	105	50
Seal oil	103	60
Salmon, any type, cooked	91	60
Coffee creamer	82	42
Butter	79	40
Chips	75	52
Moose meat	73	48
Margarine	23	50
Berries	56	24
Chicken eggs	55	41
Corn, canned	50	39
Chicken	48	41
Cereal, ready to eat	46	34
Ramen noodles	43	31

**Table 2:** Traditional foods most commonly reported on 400, 24-hour recalls among Alaska Native people (n = 177).

Traditional Food	Number of times reported	Number of people reporting
Fish other than salmon or halibut	154	80
King salmon, smoked	109	76
Agutuk	105	50
Seal oil	103	60
Salmon, any type, cooked	91	60
Moose	73	48
Berries	56	24
Tundra tea	32	19
Goose	21	12
Seal meat	20	17
Duck	16	12
Caribou	15	11
Salmon, dry	13	11
Reindeer	11	10

### QFFQ

The final QFFQ contained 150 food/beverage items under eight food categories: 14 breads and crackers; 3 cereals; 11 dairy products (including eggs); 69 meat, poultry and fish, including mixed dishes; 13 fruit, including locally gathered berries; 22 vegetables, including wild greens; 9 snacks and desserts; and 9 beverages (Table 3). Of the 150 items, 39 were traditional foods, defined as the subsistence foods consumed prior to the availability of store foods. These included moose, caribou, sea mammal meat and fat, birds, fish and other seafood, berries and wild greens.

**Table 3:** Foods and beverages on the Quantitative Food Frequency Questionnaire.

Category	Food Item
Bread and crackers(14)	Alaska fry bread; pancakes or assaliaq or waffles; pancake syrup; white bread; biscuit; whole wheat or multigrain bread; fruit bread; corn bread; pilot bread; crackers; Crisco; butter or margarine; mayonnaise; peanut butter.
Cereal(3)	Sweet cereals; low sugar cereals; oatmeal, porridge, cream of wheat, mush cooked or instant.
Dairy(11)	Low fat milk, fat free, 2% including blue box, canned or powdered; whole milk, red box, canned or powdered; chocolate milk, cocoa or hot chocolate; powdered coffee creamer; liquid coffee creamer; hard cheese; ice cream; yogurt; Cool Whip; wild bird eggs; chicken eggs.
Meat, poultry and fish(69)	Moose, caribou, reindeer or musk ox meat, fried; moose, caribou, reindeer or musk ox meat, baked, boiled, grilled, raw or frozen; moose, caribou, reindeer or musk ox meat, dried; fat or bone marrow from moose or caribou; bison or buffalo meat; bear [brown or black], baked, boiled or grilled; hamburger on a bun with condiments; ground beef or meatloaf; liver from beef; beef jerky; other beef; hotdog, sausage or corn dog, beef or pork; sausage, moose, caribou or reindeer; sandwich meat; ham meat; corned beef hash; spam or corned beef not in hash; ribs [moose, caribou or reindeer]; ribs [beef or pork]; taco; burrito; pork chops or pork roast; bacon; beaver; seal, whale or walrus, dried; seal, whale or walrus meat; seal or whale oil; seal or walrus blubber; whale skin and fat; liver from marine mammals; akutaq; wild bird, dried; wild bird, baked, boiled or grilled; fried chicken; chicken nuggets; chicken or turkey, baked, boiled or grilled; skin on poultry or bird; white fish, fried; white fish, baked, boiled, grilled, raw or frozen; white fish, dried or smoked; salmon or arctic char, fried; salmon or arctic char, baked, boiled, grilled, raw or frozen; salmon or arctic char, dried or smoked; canned salmon; blackfish; fish roe; crab; canned tuna; clams, oysters or shrimp; other fish, canned; fish sticks or nuggets; fish sticks or nuggets; moose, caribou, reindeer, or musk ox soup or stew; beef stew or chili with meat; beef or pork soup; beef stir fry with vegetables; bear [brown or black] soup or stew; bison or buffalo soup or stew; wild bird soup; chicken or turkey soup; seal, whale or walrus soup; fish soup; bean or vegetable soup; ramen noodles or cup of noodles; spaghetti or pasta without meat, including macaroni and cheese or macaroni salad; spaghetti with meat, including goulash and hamburger helper; plain rice; pizza or hot pockets; stuffing.
Fruit(13)	Wild berries; purchased berries; canned fruits in light syrup; canned fruits in syrup; apple; banana; orange, tangerine or grapefruit; honeydew melon, watermelon or cantaloupe; peach, nectarine, plum or apricot; grapes; applesauce or stewed apples; fruit salad; dried fruit.
Vegetables(22)	French fries, fried potatoes, hash browns or tater tots; baked, boiled or mashed potatoes; gravy; potato salad or cold slaw; avocado; carrots; corn; green beans or peas; cabbage or spinach; wild greens; kelp or sea weed; turnip; pumpkin, sweet potato or yams; cauliflower or broccoli; asparagus; mixed vegetables; green salad; salad dressing; refried beans; any other beans; mixed bean salad; add beans to pasta, soups or rice
Desserts and snacks(9)	Chips or popcorn; cakes or muffins; pastry, doughnut, turnover, cinnamon rolls, pop tarts; pies; cookies; granola bar, cereal bar, Rice Krispies treats; candy; pudding; nuts, trail mix or sunflower seeds
Beverages(9)	Coffee; tea; sugar or honey; artificial sweetener; sweetened drinks; unsweetened drinks; regular soda; diet soda; water

The QFFQ was designed to estimate intake over the previous 12 months. Seasonal consumption of certain foods was determined by asking if the food had been consumed throughout the year or only in certain seasons. Standard response categories were used to help participants estimate frequency of intake ranging from “never,” to “6 times or more per day.”

## Discussion

The dietary patterns showed evidence of reliance on a mix of traditional and store-bought foods. Evidence exists that a shift from traditional to store-bought foods results in decreased diet quality [5], [25]. Store-bought foods often consumed are high in energy, carbohydrate and sugar and relatively low in other nutrients. The decline in traditional food consumption among Arctic populations is associated with negative changes in health, particularly when coupled with consumption of unhealthy store-bought foods [6], [7], [10], [26]–[32]. Further decline in traditional food consumption is expected to have increasingly negative impacts.

This study developed a culturally appropriate QFFQ to estimate total intake of food and food groups to monitor the nutrition transition occurring in adult Yup'iks in the Western region of Alaska. The 150-item QFFQ will also be used to provide baseline data prior to the Food Distribution Programme on Indian Reservations (FDPIR) introduction to communities, and again after 12 months to assess the program's effect on dietary change [33]. The FDPIR, a government food assistance program, has recently been introduced to Alaska. The program was initiated in 1975 to supply food to American Indian people living on reservations who did not have access to supermarkets [33]. Foods distributed by FDPIR include canned fruit, vegetables and meats; grain products such as cereal, flour and rice; juices, dry beans, and canned and frozen meats, including ground beef and buffalo. The QFFQ described here will also be used to inform and develop a nutrition education program specific to Alaska Native people intended to prevent obesity and related chronic diseases.

A culturally specific QFFQ was necessary because of the unique diet of Alaska Native people living in Western Alaska and consists of both locally harvested traditional foods and commercially available foods. Recent studies have illustrated the significant contribution that traditional foods make to the nutritional quality of the diet, and highlight the differences between this and the standard U.S. diet [6], [7], [9], [10], [11], [31], [32]. Omitting these foods, as would occur if a standard U.S. QFFQ was used in this setting, would give a false representation of intake [20].

Use of the 24-hour recall was an important step in obtaining an accurate list of commonly consumed foods relevant to the population of interest, which is considered the most crucial step in QFFQ development [34]–[36]. The use of food models assisted participants in estimating usual amounts consumed, as recommended by Cade et al. [35].

This QFFQ, designed to capture usual intake over a previous twelve-month period, offers an advantage over short-term methods of dietary assessment, including 24-hour dietary recalls and diet records. Alaska Native peoples' diet varies significantly by season. Thus, using short-term dietary assessment methods in remote Alaskan villages would require multiple trips throughout the year, greatly increasing costs.

The present study had some limitations. The sample was predominantly female because the study targeted the primary food shoppers and preparers. Therefore, results may not be generalizable to the male population. In addition, the small sample size of the subgroups prevented us from performing subgroup analyses by the community or age group. The consumption of foods applies only to the communities we investigated, therefore our findings may not relevant to all Alaska Native communities. Exclusion of infrequently consumed food items from the QFFQ may mean that total diet was not captured; however, a longer questionnaire would have increased the burden on participants. Furthermore, the QFFQ was not validated against three 24-hour recalls, however, our previous similar studies showed a good agreement [37].

In conclusion, the 150-item QFFQ provides an up-to-date, comprehensive and unique tool for assessing dietary intake and for evaluating nutrition education programs for Alaska Native people in Western Alaska. The QFFQ will also serve to monitor the nutrition transition occurring among Yup'ik people.

## Supporting Information

Table S1 **Quantitative Food Frequency Questionnaire.**
(DOC)
