# Correction: Development of a Quantitative Food Frequency Questionnaire for Use among the Yup'ik People of Western Alaska

**DOI:** 10.1371/journal.pone.0150317

**Published:** 2016-02-22

**Authors:** The PLOS ONE Editors

Permission was not sought for listing Jennifer Johnson in the Acknowledgments for this article, and as a result, her name should not have been listed. We issue this Correction to update the Acknowledgments section, which should read as below:

“We would like to acknowledge the Yukon-Kuskokwim Health Corporation and the Norton Sound Health Corporation, and all the people who so generously gave of their time and knowledge in each community, from the clinic to the tribal council to the city office to the stores to the schools.”

We also take this opportunity to provide clarification on some additional items:

The manuscript was reviewed and approved by the Norton Sound Health Corporation (Norton Sound Research Ethics Review Board), the Alaska Native Tribal Health Consortium (ANTHC) Health Research Review Committee (HRRC) on behalf of the ANTHC Board of Directors, and the Yukon Kuskokwim Health Corporation Executive Board of Directors (and Yukon Kuskokwim Health Corporation Human Studies Committee).

It has come to the attention of the PLOS ONE Editors that a number of dietary instruments for Alaska Native populations have been published, but were not cited in the article, including:

Johnson JS, Nobmann ED, Asay E, Lanier AP. Developing a validated Alaska Native food frequency questionnaire for western Alaska, 2002–2006. Int J Circumpolar Health. 2009 Apr; 68(2):99–108. [2]

Johnson JS, Nobmann ED, Asay E. Factors related to fruit, vegetable and traditional food consumption which may affect health among Alaska Native People in Western Alaska. Int J Circumpolar Health. 2012 Mar 19;71:17345. doi: 10.3402/ijch.v71i0.17345. [3]

## References

[1] Kolahdooz F, Simeon D, Ferguson G, Sharma S (2014) Development of a Quantitative Food Frequency Questionnaire for Use among the Yup'ik People of Western Alaska. PLoS ONE 9(6): e100412 doi:10.1371/journal.pone.0100412 24963718 10.1371/journal.pone.0100412 PMC4070930

[2] Johnson JS, Nobmann ED, Asay E, Lanier AP. Developing a validated Alaska Native food frequency questionnaire for western Alaska, 2002–2006. Int J Circumpolar Health. 2009 4; 68(2):99–108 19517870 10.3402/ijch.v68i2.18319

[3] Johnson JS, Nobmann ED, Asay E. Factors related to fruit, vegetable and traditional food consumption which may affect health among Alaska Native People in Western Alaska. Int J Circumpolar Health. 2012 3 19;71:17345 doi:10.3402/ijch.v71i0.17345 22456043 10.3402/ijch.v71i0.17345 PMC3417710

