# Supplementary material for: Development of a Quantitative Food Frequency Questionnaire for Use among the Yup'ik People of Western Alaska
Source: PLoS One. 2014 Jun 25;9(6):e100412. doi: 10.1371/journal.pone.0100412 (PMC4070930; doi:10.1371/journal.pone.0100412)
Supplement: Table S1 — Quantitative Food Frequency Questionnaire. (DOC) [file pone.0100412.s001.doc]

**Table S1.** Quantitative food frequency questionnaire

| How often during the last 12 months did you USUALLY eat the following foods and how much do you USUALLY eat at one time | | In Season Only | Usual amount | Never | <1 x month | 1-3 x month | 1 x week | 2-3 x week | 4-6 x week | 1 x day |
| --- | --- | --- | --- | --- | --- | --- | --- | --- | --- | --- |
| BREADS AND CRACKERS | | | | | | | | | | |
| 1 | Any Alaskan fry bread (donut shaped and fried Assaliaq) | XXXX | A | 1 | 2 | 3 | 4 | 5 | 6 | 7 |
| 2 | Pancakes or Assaliaq, any kind (not deep fried), or waffles | XXXX | A | 1 | 2 | 3 | 4 | 5 | 6 | 7 |
| 3 | Pancake syrup, any kind | XXXX | T | 1 | 2 | 3 | 4 | 5 | 6 | 7 |
| 4 | White bread, incl. rolls, French bread, bagels, or French toast | XXXX | Sl | 1 | 2 | 3 | 4 | 5 | 6 | 7 |
| 5 | Biscuit, any kind | XXXX | # | 1 | 2 | 3 | 4 | 5 | 6 | 7 |
| 6 | Whole wheat or multi grain bread (not pantry pride) | XXXX | Sl | 1 | 2 | 3 | 4 | 5 | 6 | 7 |
| 7 | Fruit bread, banana, pumpkin, or cranberry bread | XXXX | B | 1 | 2 | 3 | 4 | 5 | 6 | 7 |
| 8 | Cornbread | XXXX | C | 1 | 2 | 3 | 4 | 5 | 6 | 7 |
| 9 | Pilot Bread | XXXX | # | 1 | 2 | 3 | 4 | 5 | 6 | 7 |
| 10 | Crackers, Royal Cremes, cheese crackers, any kind | XXXX | # crackers | 1 | 2 | 3 | 4 | 5 | 6 | 7 |
| 11 | Crisco, shortening, any kind | XXXX | Hpd Tbsp | 1 | 2 | 3 | 4 | 5 | 6 | 7 |
| 12 | Butter or margarine, any kind | XXXX | Hpd tsp | 1 | 2 | 3 | 4 | 5 | 6 | 7 |
| 13 | Mayonnaise or mayo type dressing, any kind (incl. reduced fat or light) | XXXX | Hpd tsp | 1 | 2 | 3 | 4 | 5 | 6 | 7 |
| 14 | Peanut butter, any kind | XXXX | Hpd tsp | 1 | 2 | 3 | 4 | 5 | 6 | 7 |
| CEREAL: I am going to ask you about different kinds of cereals. This is DRY cereal, before you add milk | | | | | | | | | | |
| 15 | Sweet cereals e.g. Fruit Loops, Frosted Flakes, Golden Grahams, Honey Bunches of Oats, Frosted Mini Wheats (dry only) | XXXX | D | 1 | 2 | 3 | 4 | 5 | 6 | 7 |
| 16 | Low sugar cereals, e.g. Chex, Rice Krispies, Cheerios, Raisin Bran, Wheat Bran Flakes (dry) | XXXX | D | 1 | 2 | 3 | 4 | 5 | 6 | 7 |
| 17 | Oatmeal, porridge, Cream of Wheat or mush (cooked) or instant | XXXX | D | 1 | 2 | 3 | 4 | 5 | 6 | 7 |
| DAIRY: This includes milk drunk alone, in coffee or tea, AND ON CEREAL Total per day ON DAYS USED | | | | | | | | | | |
| 18 | Low fat milk, fat free, 2%, incl. from blue box, canned, or powdered | XXXX | E | 1 | 2 | 3 | 4 | 5 | 6 | 7 |
| 19 | Whole milk, red box, or canned or powdered | XXXX | E | 1 | 2 | 3 | 4 | 5 | 6 | 7 |
